# Psychological well-being and cognitive aging in Black, Native American, and White Alzheimer’s Disease Research Center participants

**DOI:** 10.3389/fnhum.2022.924845

**Published:** 2022-07-29

**Authors:** Mary F. Wyman, Carol A. Van Hulle, Emre Umucu, Sydnee Livingston, Nickolas H. Lambrou, Fabu P. Carter, Sterling C. Johnson, Sanjay Asthana, Carey E. Gleason, Megan Zuelsdorff

**Affiliations:** ^1^W.S. Middleton Memorial Veterans Hospital, Madison, WI, United States; ^2^School of Medicine & Public Health, University of Wisconsin, Madison, WI, United States; ^3^Wisconsin Alzheimer’s Disease Research Center, University of Wisconsin, Madison, WI, United States; ^4^Department of Counseling, Educational Psychology, and Special Education, Michigan State University, Lansing, MI, United States; ^5^School of Nursing, University of Wisconsin, Madison, WI, United States

**Keywords:** psychological well-being, cognitive aging, underrepresented populations, African Americans, Native Americans

## Abstract

Psychological well-being is associated with cognition in later life but has not been examined across diverse populations—including minoritized communities at disproportionately high risk of dementia. Further, most previous work has not been able to examine links between specific facets of psychological well-being and performance within distinct cognitive domains that can capture subclinical impairment. Using a well-characterized sample followed through enrollment in an NIH-funded Alzheimer’s Disease Center, we sought to test these associations within three racial groups at baseline. Participants were *N* = 529 cognitively unimpaired Black, American Indian/Alaska Native (AI/AN), and white middle-aged and older adults (mean age = 63.6, SD = 8.1, range = 45–88 years) enrolled in the Wisconsin Alzheimer’s Disease Research Center’s Clinical Core. Predictors included validated NIH Toolbox Emotion Battery scales assessing positive affect, general life satisfaction, and meaning and purpose. Outcomes included performance on widely used tests of executive functioning and episodic memory. We conducted race-stratified regression models to assess within-group relationships. Black and AI/AN participants reported lower life satisfaction than white participants. Racial disparities were not observed for positive affect or meaning and purpose scores. Across groups, life satisfaction predicted better executive functioning. Similar associations were observed for positive affect in Black and AI/AN samples but not among whites. In general, well-being measures were not related to performance on tests of episodic memory. Our results highlight well-being as a potentially important determinant of late-life cognitive health, particularly executive functioning, that is modifiable if older adults are connected with appropriate resources and supports. Further, psychological well-being may represent a potent target for brain health interventions tailored for Black and Native communities.

## Introduction

To reduce the risk of dementia and extend the healthspan, there is increasing interest in the identification of modifiable factors that support brain health in older adults. Psychological well-being (PWB) is associated with better mental and physical health across the life course, including in late life (Steptoe et al., 2015). Psychological well-being is a construct that encompasses both; (1) affective, experiential components, e.g., experiencing happiness or relaxation, as well as feeling satisfied with life (empirically conceptualized as *hedonic* well-being); and (2) evaluative components, which include having a sense of meaning and purpose in life (conceptualized as *eudaimonic* well-being; Ryan and Deci, 2001). Hedonic and eudaimonic dimensions of well-being—feeling content “in life” vs. “with life”—appear to be distinct from one another, and each facet of well-being is associated with unique determinants (Deci and Ryan, 2008; Steptoe et al., 2015). For example, *life satisfaction* appears to be related to life stress and social relationships (Meeks and Murrell, 2001; Krause, 2004), while feeling a strong sense of *meaning and purpose* in life may be independent of emotional valence, or may even arise out of challenging life circumstances (Ryff et al., 2003).

A growing body of research is refining our understanding of the directionality between PWB and cognitive aging trajectories, moving beyond conceptualizations of cognitive impairment as a cause of diminished PWB (e.g., Wilson et al., 2013; Cho et al., 2015), to an examination of PWB as a putative protective factor (Merten et al., 2021; Bell et al., 2022). Higher levels of well-being plausibly attenuate the risk for accelerated cognitive decline or incident dementia *via* physiological and behavioral pathways, including the reduction of neuroinflammation and cardiovascular dysfunction (Allerhand et al., 2014) and the enabling of more effective emotional coping or management of medical comorbidities associated with dementia risk. In particular, recent longitudinal work has highlighted the protective role of two well-studied PWB constructs, showing that lower life satisfaction increases the 5-year risk for dementia (Peitsch et al., 2016), and purpose in life predicts incident dementia at 6–8 years (Sutin et al., 2018).

While there is evidence that individual facets of well-being variably associate with specific cognitive domains (Zahodne et al., 2014; Bell et al., 2022), many previous studies are limited by testing associations with a composite measure of PWB (e.g., Merten et al., 2021), or using clinical diagnosis or a single cognitive domain as an outcome (e.g., Gerstorf et al., 2007). This broad approach potentially conflates distinct facets and mechanisms. To obtain a more comprehensive understanding of well-being as a modifiable protective factor in preventing or delaying cognitive impairment and dementia, it is critical to examine relationships between specific facets of well-being and performance across multiple cognitive domains using validated neuropsychological tests, focusing especially on those domains likely to evidence early preclinical decline. Further, it is important to include middle-aged adults in such studies: there is growing recognition that many risk and protective factors are already highly salient by middle age or even earlier, in light of evidence that brain aging and dementia risk are shaped by exposures across the life course (Livingston et al., 2020).

Clarification of salient protective factors for cognitive aging is particularly critical within minoritized communities including Black and AI/AN older adults, who are at increased risk for dementia (Mayeda et al., 2016) but who have been historically excluded from research and its translation (Gilmore-Bykovskyi et al., 2022). Evidence does suggest that these racial disparities are not fully explained by genetic or vascular risk factors (Mayeda et al., 2016; Chen and Zissimopoulos, 2018). This is not surprising; the importance of key environmental conditions such as educational experiences in global dementia burden and comorbid precursors such as diabetes has been well-documented (Oghagbon and Giménez-Llort, 2019; Livingston et al., 2020; Oghagbon et al., 2022). In the U.S., racial disparities in cognitive aging appear to be driven in large part by inequitably distributed social determinants of health including educational experiences and wealth (Sisco et al., 2015; Mayeda et al., 2016; Chen and Zissimopoulos, 2018) as well as resultant lifetime stress exposures (Zahodne et al., 2019; Zuelsdorff et al., 2020; Chen et al., 2022). These upstream social factors may operate in part through cumulative impacts on personal well-being (Krause, 2004). Inclusive studies that explore downstream protective factors such as well-being can offer crucial evidence for additional, later-life intervention loci. However, the literature on late-life well-being and cognition is generally lacking in diversity, with many studies using overwhelmingly white samples, combining racial groups into a “non-white” category, or not reporting participant race (e.g., Peitsch et al., 2016; Sutin et al., 2018; Merten et al., 2021). Data from American Indian or Alaska Native (AI/AN) samples are near absent. As a result, understanding of specific facets of PWB as a modifiable protective factor in minoritized older adult populations—and thus a potential target for culturally tailored interventions—is limited, despite evidence demonstrating social disparities in levels of well-being (Skarupski et al., 2013). Because historical and current discrimination associated with minoritization systematically impacts lived experiences in these same underrepresented communities, shaping both risk and protective exposures, it is a priority to establish population-specific distributions and magnitudes of effect for well-being and cognitive health.

To begin addressing these gaps, we aimed to examine PWB as a predictor of cognitive health, drawing from a well-characterized cohort of AI/AN, Black, and white middle-aged and older adults enrolled in a longitudinal prospective study. Using recently developed measures from the NIH Toolbox, we examined associations between three distinct facets of hedonic and eudaimonic well-being and contemporaneous performance on validated tests of executive function and episodic memory. We focused on these measures, which capture hippocampally-based learning as well as some aspects of the complex domain of the executive function, based on their sensitivity to early neurotransmitter and substrate changes (Lezak et al., 2012; Anderson et al., 2016). We hypothesized that there would be a positive association between facets of PWB and cognitive performance, especially executive function, across the three samples.

## Materials and Methods

### Sample and procedure

Participants are enrolled in the Wisconsin Alzheimer’s Disease Research Center (WI ADRC) Clinical Core, a longitudinal study of brain aging and cognitive health. WI ADRC enrollees undergo comprehensive annual or biennial evaluations, involving neuropsychological testing, a physical exam and collection of samples for laboratory assays, and self-reported health and lifestyle data. Based on these evaluations, cognitive status is determined through interdisciplinary expert consensus discussion. The study was approved by the University of Wisconsin Institutional Review Board.

The study sample included participants with complete predictor and outcome data for at least one study visit, who were aged ≥45 years and had unimpaired cognition (no Mild Cognitive Impairment or dementia at that visit and ≥1 preceding or subsequent visits). Race was self-identified by participants at the initial ADRC study visit using the following categories, which are part of uniform data collection standardized across all ADRC sites: white, Black or African American, American Indian or Alaska Native (AI/AN), Native Hawaiian or Pacific Islander, Asian, or a write-in option. The analytic sample included all participants who endorsed primary race as white, Black or African American (in this article, we also use the term “Black”), or AI/AN (here, we also use the term “Native American”), totaling 529 participants.

Measures of well-being were added to the WI ADRC study protocol in 2017. WI ADRC data collection was paused in March 2020 due to preemptive institutional restrictions to minimize transmission of the novel coronavirus. As a result, all data in this study were drawn from visits occurring after the 2017 protocol modification and prior to COVID-19 pandemic-related shutdowns.

### Measures

#### Psychological well-being

Psychological well-being was assessed with scales from the NIH Toolbox iPad version 2.0, constructed and normed with consideration of geriatric and culturally diverse populations (Victorson et al., 2013). Drawing items from validated existing scales, these measures utilize item response theory-based administration to minimize participant burden and enable precise assessment of each construct (Salsman et al., 2014). Key predictors in this study included two scales of hedonic well-being, Positive Affect (possible 34 items, e.g., “I felt cheerful”; reported for past 7 days) and General Life Satisfaction (possible 10 items, e.g., “My life is going well”; no time interval specified); we measured a facet of eudaimonic well-being with the Meaning and Purpose scale (possible 18 items, e.g., “My life has a clear sense of purpose”; no time interval specified). Per NIH Toolbox guidance, our analyses used t-scores calculated using a nationally representative normative sample (Salsman et al., 2014).

#### Cognitive function

Outcomes were assessed with four measures of episodic memory and executive functioning. Immediate memory was assessed using the sum of trials 1–5 of the Rey Auditory and Verbal Learning Test (RAVLT), and RAVLT total recall score, after approximately 20 min delay, reflected delayed memory (Schmidt, 1996). Processing speed and mental flexibility, two dimensions of executive functioning, were assessed with completion time on the Trail Making Test, parts A and B, respectively (TMT; Reitan, 1992). For these measures, higher scores indicate slower speed and therefore poorer performance.

#### Covariates

Model covariates included demographic variables empirically associated with cognitive test performance and with psychological well-being. Age in years at the study visit was calculated from birth month and year. Prior to modeling, years of education were categorized as a high school diploma or less, some college, bachelor’s degree, or more than a bachelor’s degree. Gender was self-reported as either male or female (non-binary options were not queried in the uniform data set questionnaires utilized by the ADRCs).

### Analysis

Stratification by race and within-group analyses were utilized to examine associations for several reasons. Importantly, experiences of minoritization shape life-spanning conditions and exposures that differ by race in myriad ways, with unique implications for psychosocial risk and resilience. Within-group analyses highlight heterogeneity within populations and identify protective factors likely to have community-specific distributions, inputs, and clinical implications (Whitfield et al., 2008). Additionally, previous work has demonstrated that pathways to Alzheimer’s Disease Research Center study participation can differ significantly by race in complex and often unobservable ways, potentially creating measurable and unmeasurable selection-related biases in combined samples (Gleason et al., 2019). In the WI ADRC, for example, Black and AI/AN participants are typically enrolled through community-engaged outreach initiatives, while white participants are more likely to be referred by providers in memory clinics or through passive processes such as word-of-mouth within affected families. This may result in the recruitment of an overall healthier sample representing some racial groups and an overall more cognitively vulnerable sample from others.

Descriptive statistics were calculated for the combined sample and each race group, and a chi-square test or Kruskal-Wallis test, as appropriate, was applied for group comparisons. Within each race group, linear regression models were then conducted for each predictor and outcome, adjusted for demographic covariates. Although well-being measures were non-normally distributed within race groups, regression diagnostics indicated that there was no substantial bias in residuals. Using Cook’s D for models fit to Black and white samples, no influential outliers were detected (due to the small sample size, we did not check model diagnostics for AI/AN participants). Aligning with growing calls from methodologists in multiple disciplines (Karpen, 2017; Wasserstein et al., 2019; Butler, 2021), we interpret model results primarily through inspection of 95% confidence intervals for asymmetry and direction, rather than relying solely on the determination of statistical significance.

## Results

**Figure 1:**
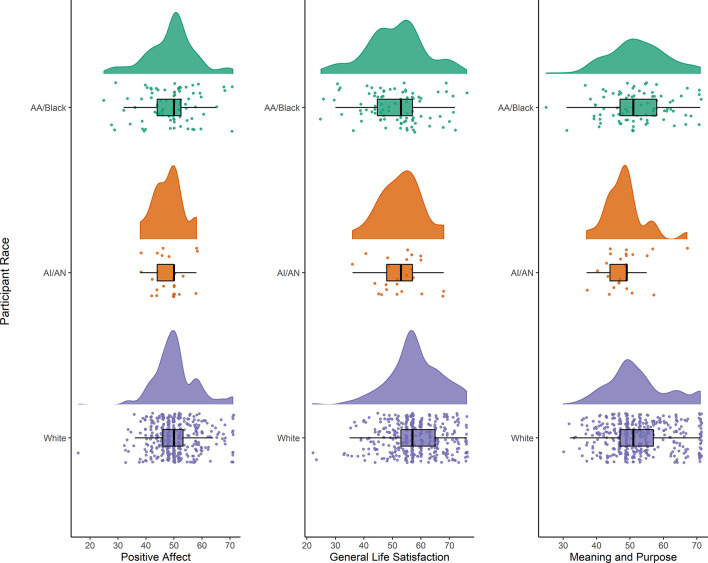
Raincloud plot showing the distribution of three scales assessing facets of psychological well-being from the NIH Toolbox, in African American/Black (*N* = 88), American Indian/Alaska Native (*N* = 25), and white (*N* = 416) subsamples. Within each figure, the cloud depicts the distribution within that race group, the box and whiskers show the median, interquartile range, and 1.5 times the interquartile range above the upper quartile and below the lower quartile. Drops represent individual responses.

The combined sample was predominantly female, with an average age of 63.6 years (SD 8.1). AI/AN participants were older, and Black and AI/AN participants reported less formal education than their white peers (see Table 1). Significant differences across groups were noted for all cognitive outcomes, with the white sample averaging the highest unadjusted performance across measures. No racial differences were observed in mean levels of positive affect. However, Black and AI/AN participants endorsed lower life satisfaction compared to white participants (mean t-scores = 51.1, 52.9, and 58.0 respectively; *H*_(2)_ = 39.5, *p* < 0.001). AI/AN participants reported less meaning and purpose than Black or white participants (mean t-scores = 48.0, 52.3, and 52.4 respectively; *H*_(2)_ = 6.2, *p* = 0.04). For all three facets of well-being, there was substantial overlap in the distribution of scores across racial groups. We presented a detailed visualization of these distributions in Figure 1.

**Table 1:** Sample characteristics (*N* = 529).

	**Combined sample, range and frequencies**	**African American/Black**	**American Indian/Alaska Native**	**White**	***p*-value**
	*N* = 529	*N* = 88	*N* = 25	*N* = 416	
Age, years, M (SD)	457–88	64.2 (9.0)	68.2 (8.3)	63.1 (7.7)	0.004
Female, N (%)		61 (69%)	20 (80%)	266 (64%)	0.19
Educational attainment, M (SD)	8–20 years	15.1 (2.5)	16.0 (2.3)	16.6 (2.2)	<0.001
≤HS diploma, N (%)	37 (7.0)	14 (15.9)	1 (4.0)	22 (5.3)
some college/post-HS, N (%)	116 (21.9)	34 (38.6)	8 (32.0)	74 (17.8)
Bachelor’s degree, N (%)	149 (28.2)	15 (17.1)	6 (24.0)	128 (30.8)
>Bachelor’s degree, N (%)	227 (42.9)	25 (28.4)	10 (40.0)	192 (46.2)
Positive Affect, M (SD)	16–71	48.9 (9.0)	47.8 (5.6)	50.4 (7.5)	0.26
General Life Satisfaction, M (SD)	22–76	51.1 (10.7)	52.9 (7.6)	58.0 (9.3)	<0.001
Meaning and Purpose, M (SD)	25–71	52.3 (9.2)	48.0 (5.5)	52.4 (9.2)	0.04
RAVLT 1–5, M (SD)	20–75	42.6 (9.1)	41.3 (8.2)	51.8 (10.3)	<0.001
RAVLT Delayed, M (SD)	0–15	7.8 (3.3)	7.4 (3.0)	10.2 (3.0)	<0.001
Trail Making Test, Part A (seconds), M (SD)	10–97	35.8 (16.2)	31.9 (11.5)	23.1 (7.5)	<0.001
Trail Making Test, Part B (seconds), M (SD)	19–300	104.2 (60.9)	92.2 (34.5)	55.7 (22.5)	<0.001

**Note**: Scores on Positive Affect, General Life Satisfaction, and Meaning and Purpose represent t-scores calculated using a nationally representative normative sample (see Salsman et al., 2014). RAVLT, Rey Verbal and auditory Learning Test. The difference across groups was tested using a chi-square test for categorical variables or the Kruskal-Wallis test for continuous variables.

**Figure 2:**
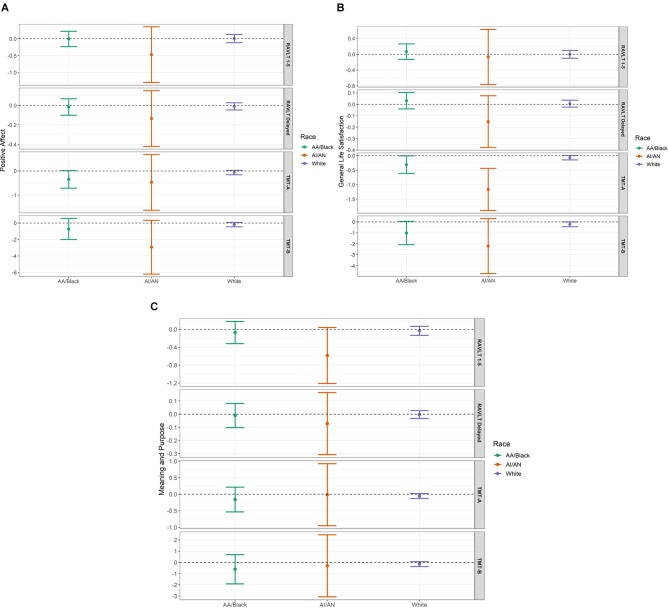
**(A–C)** The three panels **(A)** (Positive Affect), **(B)** (General Life Satisfaction), and **C** (Meaning and Purpose) show visualizations of linear regression estimates and 95% confidence intervals for each predictor and outcome, stratified by subsample: African American/Black (*N* = 88), American Indian/Alaska Native (AI/AN, *N* = 25), and White (*N* = 416). All models are adjusted for age, gender, and educational attainment. RAVLT, Rey Verbal and Auditory Learning Test; TMT, Trail Making Test. Scores on the Trail Making Test, parts **(A and B)** reflect time to task completion, such that lower scores indicate better performance.

Linear regression modeling revealed unique demographic-adjusted associations of the three dimensions of well-being with domains of cognitive performance. For all models stratified by race group, we present estimates, probability values, and confidence intervals in Table 2; to facilitate interpretation of our findings, Figures 2A–C show visualizations of the model for each predictor. As can be seen here, precision was limited for Black and AI/AN participants due to sample size. Across groups, positive affect did not associate with immediate or delayed memory (RAVLT 1–5 and RAVLT Delayed scores, respectively). In the Black sample, there was a modest association between positive affect t-score and TMT-A time to completion, such that higher levels of positive affect predicted faster processing speed (unstandardized *b* = −0.35, 95% CI [−0.71, 0.02]). In AI/AN participants, we found a similar association between positive affect and mental flexibility, as reflected in performance on the TMT-B (*b* = −2.94, 95% CI [−6.21, 0.33]). No such associations were observed in the white sample.

**Table 2:** Linear regression: relationships between measures of psychological well-being and cognitive test performance by race.

	**RAVLT 1–5**	**RAVLT Delayed**	**Trail Making Test A**	**Trail Making Test B**
	**Displayed are the unstandardized coefficients, (95% confidence interval), *p*-value**			
**Positive Affect**
AA/Black	−0.01 (−0.23, 0.22), 0.97	−0.02 (−0.10, 0.07), 0.70	**−0.35 (−0.71, 0.02), 0.06**	−0.72 (−2.00, 0.56), 0.27
AI/AN	−0.47 (−1.29, 0.36), 0.25	−0.13 (−0.42, 0.15), 0.34	−0.47 (−1.63, 0.68), 0.40	**−2.94 (−6.21, 0.33), 0.08**
White	0.01 (−0.12, 0.13), 0.82	−0.01 (−0.04, 0.03), 0.60	−0.06 (−0.16, 0.03), 0.18	−0.19 (−0.46, 0.14), 0.17
**General Life Satisfaction**
AA/Black	0.07 (−0.13, 0.26), 0.49	0.03 (−0.04, 0.10), 0.39	**−0.31 (−0.60, −0.003), 0.05**	**−1.00 (−2.07, 0.05), 0.06**
AI/AN	−0.07 (−0.76, 0.63), 0.84	−0.15 (−0.38, 0.08), 0.18	**−1.16 (−1.89, −0.43), 0.004**	**−2.20 (−4.71, 0.31), 0.08**
White	−0.001 (−0.10, 0.10), 0.98	0.006 (−0.02, 0.04), 0.70	**−0.07 (−0.14, 0.01), 0.08**	**−0.22 (−0.43, 0.003), 0.05**
**Meaning and Purpose**
AA/Black	−0.07 (−0.32, 0.18), 0.56	−0.01 (−0.10, 0.08), 0.82	−0.16 (−0.53, 0.22), 0.40	−0.60 (−1.91, 0.69), 0.36
AI/AN	**−0.58 (−1.21, 0.04), 0.07**	0.001 (−0.16, 0.17), 0.98	0.46 (−0.21, 1.13), 0.17	−0.03 (−2.18, 2.12), 0.98
White	−0.02 (−0.13, 0.08), 0.66	0.003 (−0.03, 0.03), 0.87	−0.06 (−0.13, 0.02), 0.14	−0.15 (−0.41, 0.11), 0.25

Notes. African American (AA)/Black, *N* = 88; American Indian/Alaska Native (AI/AN), *N* = 25; White, *N* = 416. All models adjusted for age, gender, and educational attainment. RAVLT, Rey Verbal and Auditory Learning Test. Scores on the Trail Making Test, parts A and B reflect time to task completion, such that *lower* scores indicate better performance. Stratified by race, R^2^ ranges are 0.04–.30 (AA/Black), 0.20–.51 (AI/AN) and 0.11–.18 (White). **Bolded** cells indicate potentially meaningful associations as interpreted through inspection of confidence intervals for asymmetry and direction (Karpen, 2017; Wasserstein et al., 2019; Butler, 2021).

Across groups, life satisfaction was not predictive of immediate nor delayed memory scores. However, greater life satisfaction is associated with better performance in both measures of executive functioning across all three groups.

In general, meaning and purpose did not associate with cognitive test performance. An exception was observed in the AI/AN sample, wherein higher meaning and purpose t-scores were marginally associated with poorer immediate memory function (*b* = −0.58, 95% CI [−1.21, 0.04]). This relationship was robust to the removal of potentially influential outliers in the AI/AN sample (data not shown).

## Discussion

In community-dwelling volunteer samples of Black, AI/AN, and white cognitively intact older adults, using validated measures of well-being and objective cognitive testing, we found that greater life satisfaction predicted better performance on tests of executive function, but not memory. Similar positive associations were observed for positive affect in Black and AI/AN participants. Overall, our results point to these facets of hedonic well-being as potentially modifiable protective factors or markers for late-life cognitive health in two racial groups that have been historically excluded from research despite disproportionately high risk for cognitive impairment and dementia. Feelings of life satisfaction and positive affect can be, as noted earlier, readily influenced by upstream conditions—including lessening by stress exposure and improvement in the presence of positive social relationships. If future work replicates findings from our Black and AI/AN samples, the importance of illuminating and modifying community-specific determinants of well-being is critical.

Our finding that Black and AI/AN participants reported lower life satisfaction than white peers reaffirms previously observed racial disparities in this aspect of well-being (Barger et al., 2009; Skarupski et al., 2013; Zhang et al., 2017). This body of evidence suggests that the distribution and salience of individual- and community-level conditions that contribute to life satisfaction are quite population-specific, varying as a marker of racialized social histories and inequities. In our sample, for instance, education levels were relatively high across racial groups. Previous work indicates that education and income show linear positive associations with life satisfaction in whites, but a non-linear or U-shaped association in Black populations (Zhang et al., 2017). Those authors proposed that this may reflect the reality that educational achievements do not yield expected economic returns for many Black adults, subsequently leading to more negative evaluations of one’s life experience. Disparities in key determinants of well-being—e.g., medical comorbidities, accessible emotional support, and experiences of race-based discrimination—also partially account for Black-white differences in life satisfaction (Barger et al., 2009; Paradies et al., 2015). Racial-ethnic comparison studies of psychological well-being that include Native samples are rare. However, while the culture and experiences of Black and Native American populations in the U.S. are clearly distinct, they share population-level burdens of historical trauma (Whitbeck et al., 2004), institutional and interpersonal discrimination (Findling et al., 2019), and resultant chronic disease (Hutchinson and Shin, 2014; Warne and Lajimodiere, 2015). Our finding that racial disparity in life satisfaction extended to an AI/AN sample is unsurprising.

In contrast to findings for life satisfaction, distributions of positive affect did not significantly differ by race in the present study. Previous findings in this area have been mixed. A study using data from the Midlife in the U.S. (MIDUS) study examined the distribution of psychological assets across race and found that Black adults reported significantly higher positive affect than their white peers (Boehm et al., 2015). In the population-based Washington Heights-Inwood Community Aging Project (WHICAP) cohort, Zahodne and colleagues found no race differences in positive affect (Zahodne et al., 2018). They did find that Black participants scored higher on measures of life purpose compared to their white peers, a difference that has also been observed in MIDUS (Ryff et al., 2003), while in the current study the AI/AN sample reported lower life purpose on the Emotion Toolbox measure than Black or white participants. These unexpected findings may reflect sampling-related differences in the participants who chose to enroll in the parent ADRC cohort, or cultural differences in response to the specific items included. Positive affect is an “emotional” rather than “evaluative” aspect of well-being, and like life purpose has been shown to increase throughout later life (Kieny et al., 2020). Therefore, it is possible that between-group age differences in our study, and between our sample and others, account for discordance in findings.

In regression models, life satisfaction was related to cognitive outcomes across groups. This finding adds to a growing body of work in predominantly white samples demonstrating relationships between this domain of hedonic well-being and cognitive outcomes in older age, including the rate of decline over time (Gerstorf et al., 2007) and incident dementia (Peitsch et al., 2016). Drawing from the nationally representative and population-based Health and Retirement Study, Sutin and colleagues found that associations between life satisfaction and risk for dementia were attenuated by adjustment for depressive symptoms (Sutin et al., 2018). This is consistent with life course research suggesting that mental disorder is both upstream and downstream of life satisfaction (Fergusson et al., 2015). In our study, the emotive measure of hedonic well-being, positive affect, showed modest associations with executive function in Black and AI/AN samples but not in whites. This finding echoes recent research among older Mexican Americans, another minoritized community in the U.S., linking positive affect to cognitive health over time (Castro-Schilo et al., 2019).

We did not find a clear relationship between our measure of eudaimonic well-being, life meaning and purpose, and the measured cognitive outcomes. This unexpected finding is similar to results from a study exploring NIH Toolbox-derived meaning and purpose scores in Black and white samples in WHICAP (Zahodne et al., 2018), but is in contrast to some prior work in this area. Using data from a Midwestern volunteer cohort, Boyle and colleagues found that greater life purpose associates with reduced risk for cognitive decline (Boyle et al., 2010), delaying dementia onset by as much as 6 years (Boyle et al., 2022), possibly *via* greater functional resilience to accumulating brain pathologies (Boyle et al., 2012). Our study did find an association of greater meaning and purpose with poorer memory performance in the AI/AN sample, similar to WHICAP findings for participants reporting Hispanic ethnicity. This may reflect the presence of unmeasured confounders: Zahodne et al. (2018) note that a sense of meaning can develop from personal or health challenges that also increase the risk for impaired cognitive performance. Alternately, our findings may indicate issues with the validity of this scale in Native American populations. Even in nationally representative samples, Native American participants are often missing: in the NIH Toolbox Emotion Battery development process, there were no persons identifying as AI/AN in the testing sample (Salsman et al., 2014). Well-being researchers have noted that cultural context and values may impact responses on a self-report questionnaire, even when the construct remains important to health and other outcomes (Deci and Ryan, 2008).

Finally, debates in the literature highlight the contested relevance of different dimensions of well-being (e.g., hedonic vs. eudaimonic; Deci and Ryan, 2008) and the ongoing challenges in reliable measurement (Steptoe et al., 2015). As currently conceptualized, eudaimonia is the experience of “living well” and with high character, independent of circumstances or how one feels (Ryan et al., 2008). While our results suggest that distinctions between these dimensions may be significant for the identification of risk and protective factors for cognitive health in later life, mixed findings point to the critical need for continued clarification of these constructs and their relevance to health across diverse populations, particularly those who have been historically excluded from research.

Robust associations of well-being measures were seen only for the cognitive functions of processing speed (TMT-A) and to some extent mental flexibility (TMT-B), but not for episodic memory. These findings are consistent with our team’s prior work examining cognition and psychosocial factors including perceived social support (Zuelsdorff et al., 2013) and stressful life events (Zuelsdorff et al., 2013, 2020). It is possible that this, in part, reflects the inclusion of middle-aged and “young-old” participants across these studies, as changes in speed and executive function have been shown to mediate the relationship between chronological age and changes in episodic memory (Lee et al., 2012).

The nature of our sample and other study limitations deserve discussion. First, the small size of our Black and AI/AN samples limited not only precision but mechanistic modeling potential. The use of diverse cohorts that are adequately powered for pathway analysis can help parse whether important mental and physical health correlates, and protective factors like social support and social embeddedness (Cho et al., 2015), represent mediators or confounding variables in models of well-being and cognition. Personal characteristics that intersect with a race to create unique risk and protective factors for mental and cognitive health, such as gender (Brave Heart et al., 2016), are also critical to consider. In particular, the associations between gender and well-being, and gender and cognitive performance, are highly complex—especially across middle-aged and older cohorts. There is a pressing need for more research in this area. While our limited sample sizes and overrepresentation of women preclude meaningful examination of gender in our primary within-race analyses, for interested readers, we include gender-stratified descriptive data on outcomes and predictors in the supplementary material (see Supplementary Table). Our small sample sizes similarly limited our ability to assess the potential role of cohort effects on the relationships between well-being and cognitive outcomes, despite evidence for cohort differentials in both self-reported well-being (Sutin et al., 2013) and in cognitive impairment and dementia (Weuve et al., 2018). Future studies in larger similarly middle-aged and older samples should explore potentially distinct age, period, and cohort effects as they relate to well-being and cognitive aging. Second, while our outcomes were specifically selected based on their sensitivity to Alzheimer’s Disease-related cognitive changes (Lezak et al., 2012; Anderson et al., 2016), our measures reflect only two aspects of the complex cognitive domain of executive functioning. Future work should include examining associations of well-being with other cognitive domains and additional, nuanced measures of executive functioning changes. Further, the ADRC-based cohort is not representative of the general older adult population. Despite notable findings for community-specific relationships between well-being and cognitive health for Hispanic/Latino populations (Zahodne et al., 2018; Castro-Schilo et al., 2019) and growing efforts prioritizing engagement with and inclusion of Hispanic/Latino communities in our ADRC, we were not able to model the importance of Hispanic ethnicity in this study. And, while the use of community-engaged outreach to recruit heterogeneous Black and AI/AN samples into the WI ADRC is a strength of the study, white participants are much more likely to enroll following referral from a memory clinic. As a result, their risk profiles may be unique: they possess many “healthy volunteer” protective exposures including high educational attainment, but also an underlying propensity for cognitive impairment (Gleason et al., 2019). While our stratified analytic approach preempts issues of internal validity stemming from selection bias tied to race and recruitment, external validity may be limited. In addition, because all models were selected *a priori* based on strong theoretical and empirical bases, we did not correct for multiple comparisons (Rothman, 1990) but the possibility of Type I error affecting some statistical results cannot be excluded. This concern is mitigated by the overall consistency of our findings. Finally, and crucially, these cross-sectional data preclude definitive determination of the temporal relationship between predictor and outcome. Indeed, evidence suggests that the relationship between well-being and cognitive health is likely bidirectional (Allerhand et al., 2014). Though the use of a well-characterized, younger sample and the exclusion of participants with clinical diagnoses partially address this concern, the dementia prodrome is long (Livingston et al., 2020). Levels of well-being may be impacted by pre-clinical neurodegenerative changes, not yet resulting in detectable cognitive impairment (Babulal et al., 2016). Longitudinal studies spanning adult life and exploring mediating mechanisms, as well as intervention studies targeting well-being and examining the subsequent impact on cognition, will be critical in order to establish the causality of these relationships.

Nonetheless, our study makes several unique contributions. First, we include a well-characterized sample of persons identifying as AI/AN. These populations experience a disproportionately high dementia burden (Mayeda et al., 2016) but represent an “invisibility minority” in cognitive aging research, with very few studies examining cognitive risk profiles specific to Native American communities. The AI/AN sample in the current study is small, and the WI ADRC participants cannot represent the diverse histories and cultures of hundreds of Native American tribes. However, preliminary estimates in this study represent an important step toward characterizing modifiable, clinically relevant psychosocial correlates of cognitive health in regional Native American older adults and complement substantive existing frameworking and assessment work on Indigenous wellness and resilience (Hodge et al., 2009; Dirks, 2016; Oré et al., 2016; Rountree and Smith, 2016). We also go beyond previous work focused exclusively on participants in late life; importantly, by including middle-aged adults in our examination of cognitive health, we contribute to the life course literature on cognitive aging. Because dementia pathology begins decades before the onset of symptoms, it is important to understand the relationships between well-being and cognition early in this hypothesized trajectory. Finally, and most broadly, our work adds to emerging research conceptualizing well-being as a modifiable protective factor that can support cognitive health and delay decline.

Taken as a whole, findings on the relationship between facets of well-being and cognition suggest that diverse relevant mechanisms for these associations exist. How these associations may vary across racialized populations in the US, and across personal characteristics that intersect with race, such as gender, has not been examined in-depth. In terms of clinical utility to support successful aging among diverse communities, therefore, there may be multiple potential foci for intervention. Research prioritizing the inclusion of previously underrepresented communities is needed to guide public health interventions to address increased dementia risk.

Our findings provide evidence for a robust relationship between facets of hedonic well-being and cognitive health across three racial groups. Our results underscore the importance of continued research in this area, both to replicate these findings in larger samples of historically excluded populations and to identify community-specific determinants of well-being that can be subsequently targeted through clinical intervention and public health policy. Studies have demonstrated a strong role of social support, spirituality, and enculturation in fostering life satisfaction and other measures of well-being, particularly in racialized populations including Black and Native American communities (Hodge and Nandy, 2011; Roh et al., 2015; Yoo et al., 2018). Well-established structural inequalities, including reduced access to culturally competent medical and mental health care (Gone and Trimble, 2012; Garrett et al., 2015), are likely key determinants of well-being and should be an area of attention for clinicians caring for older adults. Interventions to improve well-being may target the wrong need if they are not based on strong community-specific evidence, as exemplified by recent work suggesting that Black but not white caregivers of older adults report gains in psychological and emotional well-being associated with caregiving activities, even as they reported more caregiving-related financial difficulties than white peers (Fabius et al., 2020). And importantly, interventions to optimize well-being may be most impactful for older adults already at risk (Bartholomaeus et al., 2019). Successful well-being interventions may also have multiplicative effects on other established risk factors for cognitive impairment and accelerated decline, such as poor physical and mental health. Health systems should collaborate with local organizations to identify and link patients with existing community assets and resources to support continued social and spiritual connections (Wildman et al., 2019).

In summary, our findings contribute to the sparse literature on psychological well-being and cognition in minoritized older adults and provide additional evidence for the clinical utility of using community-tailored interventions that promote well-being to help reduce dementia-related health disparities in these populations. Recent evidence suggests that at least 40% of the global burden of dementia is attributable to modifiable risk factors (Livingston et al., 2020). Timely identification of modifiable sources of cognitive resilience, and their promotion and facilitation through practice and policy, is crucial for addressing the current dementia crisis—particularly its outsized burden on minoritized communities (Mayeda et al., 2016). Psychological well-being may represent an important nexus of protection that can disrupt multiple pathways of risk. Future work among racially, ethnically, and geographically diverse cohorts can be harnessed to help improve the cognitive health of all older adults and achieve equity for historically excluded and underserved communities.

## Data Availability Statement

The raw data supporting the conclusions of this article will be made available by the authors, without undue reservation.

## Ethics Statement

The studies involving human participants were reviewed and approved by University of Wisconsin Institutional Review Board. The patients/participants provided their written informed consent to participate in this study.

## Author Contributions

MW, CVH, EU, MZ, and CG were responsible for study design, analyses, and manuscript drafting. SL, NL, FC, SJ, and SA made contributions to data acquisition and revising the manuscript. All authors agree to be accountable for the content of the work. All authors contributed to the article and approved the submitted version.

## Funding

This work was supported with resources and the use of facilities at the Geriatric Research, Education, and Clinical Center at the W.S. Middleton Memorial Veterans Hospital, Madison, WI (Manuscript # 009-2021). MW is supported under a Career Development Award [IK2 HX003080] funded by the U.S. Department of Veterans Affairs Health Services Research & Development Service, VA Office of Research & Development. Additional funding came from the Alzheimer’s Association [AARF-18-562958, PI MZ, PhD] and the National Institute on Aging-National Institutes of Health [R01 AG054059, PI CG, PhD; R03 AG063303, PI MZ, PhD; and P30 AG062715, PI SA, MD].
