# Supplementary material for: Psychological well-being and cognitive aging in Black, Native American, and White Alzheimer’s Disease Research Center participants
Source: Front Hum Neurosci. 2022 Jul 29;16:924845. doi: 10.3389/fnhum.2022.924845 (PMC9372578; doi:10.3389/fnhum.2022.924845)
Supplement: Supplementary file 1 [file Table_1.docx]

**Supplementary Table.** Outcomes and predictors by race and self-reported gender.

|  | **AA/Black** | | **AI/AN** | | **White** | |
| --- | --- | --- | --- | --- | --- | --- |
|  | **Female (N=61)** | **Male (N=27)** | **Female (N=20)** | **Male (N=5)** | **Female (N=266)** | **Male (N=150)** |
| **Positive Affect** | | |  |  |  |  |
| Mean (SD) | 48.7 (8.5) | 49.4 (10.2) | 47.5 (4.64) | 50.0 (8.48) | 50.7 (8.00) | 49.9 (6.63) |
| Range | 29.0 - 71.0 | 25.0 - 71.0 | 38.0 - 58.0 | 38.0 - 58.0 | 16.0 - 71.0 | 33.0 - 71.0 |
| **Life Satisfaction** | | |  |  |  |  |
| Mean (SD) | 50.8 (10.6) | 51.6 (11.0) | 51.4 (6.0) | 58.8 (11.1) | 57.9 (9.7) | 58.1 (8.5) |
| Range | 25.0 - 76.0 | 26.0 - 70.0 | 36.0 - 60.0 | 41.0 - 68.0 | 22.0 - 76.0 | 33.0 - 76.0 |
| **Meaning and Purpose** | | |  |  |  |  |
| Mean (SD) | 51.9 (9.25) | 53.2 (9.05) | 47.5 (4.6) | 51.6 (10.2) | 52.6 (9.4) | 52.1 (8.75) |
| Range | 25.0 - 71.0 | 31.0 - 71.0 | 37.0 - 57.0 | 43.0 - 67.0 | 32.0 - 71.0 | 30.0 - 71.0 |
| **RAVLT 1-5** |  |  |  |  |  |  |
| Mean (SD) | 43.3 (9.00) | 41.1 (9.4) | 40.4 (7.85) | 44.8 (9.31) | 54.6 (9.39) | 47.0 (10.0) |
| Range | 21.0 - 65.0 | 23.0 - 64.0 | 25.0 - 57.0 | 34.0 - 55.0 | 24.0 - 75.0 | 20.0 - 75.0 |
| **RAVLT Delayed** | | |  |  |  |  |
| Mean (SD) | 8.04 (3.16) | 7.42 (3.48) | 6.95 (2.82) | 9.00 (3.32) | 10.85 (2.82) | 9.06 (3.03) |
| Range | 1.00 - 15.0 | 0.00 - 14.0 | 2.00 - 14.0 | 4.00 - 13.0 | 2.00 - 15.0 | 0.00 - 15.0 |
| **TMT-A** |  |  |  |  |  |  |
| Mean (SD) | 35.4 (16.2) | 36.6 (16.3) | 31.8 (11.6) | 32.2 (12.4) | 22.4 (7.34) | 24.2 (7.66) |
| Range | 12.0 - 97.0 | 18.0 - 72.0 | 16.0 - 61.0 | 20.0 - 51.0 | 10.0 - 54.0 | 11.0 - 55.0 |
| **TMT-B** |  |  |  |  |  |  |
| Mean (SD) | 106.4 (64.7) | 99.2 (52.0) | 96.1 (37.1) | 76.4 (14.3) | 55.5 (24.7) | 55.9 (18.3) |
| Range | 37.0 - 300.0 | 34.0 - 223.0 | 39.0 - 180.0 | 64.0 - 101.0 | 19.0 - 292.0 | 24.0 - 132.0 |

Notes. RAVLT=Rey Verbal and Auditory Learning Test; TMT=Trail Making Test, parts A & B. Some participants did not complete the RAVLT test, leading to smaller Ns for certain groups: AA/Black females, 6, AA/black males, 1; AI/AN females, 1; White females, 6; white males, 2. Scores on TMT-A and TMT-B reflect time to task completion, such that lower scores indicate better performance.
